# Gene array analysis of neural crest cells identifies transcription factors necessary for direct conversion of embryonic fibroblasts into neural crest cells

**DOI:** 10.1242/bio.015735

**Published:** 2016-02-12

**Authors:** Tsutomu Motohashi, Natsuki Watanabe, Masahiro Nishioka, Yuhki Nakatake, Piao Yulan, Hiromi Mochizuki, Yoshifumi Kawamura, Minoru S. H. Ko, Naoki Goshima, Takahiro Kunisada

**Affiliations:** 1 Department of Tissue and Organ Development, Regeneration, and Advanced Medical Science, Gifu University Graduate School of Medicine, Gifu 501-1194, Japan; 2 Japan Science and Technology Agency (JST), Core Research for Evolutional Science and Technology (CREST), Tokyo 102-0076, Japan; 3 Laboratory of Genetics, National Institute on Aging, NIH, Baltimore, MD 21224, USA; 4 Department of Systems Medicine, Sakaguchi Laboratory, Keio University School of Medicine, Tokyo 160-8582, Japan; 5 Japan Biological Informatics Consortium (JBiC), Tokyo 135-8073, Japan; 6 Molecular Profiling Research Center for Drug Discovery, National Institute of Advanced Industrial Science and Technology, Tokyo 135-0064, Japan

**Keywords:** Neural crest, Mouse embryonic fibroblasts, Sox10, Direct conversion

## Abstract

Neural crest cells (NC cells) are multipotent cells that emerge from the edge of the neural folds and migrate throughout the developing embryo. Although the gene regulatory network for generation of NC cells has been elucidated in detail, it has not been revealed which of the factors in the network are pivotal to directing NC identity. In this study we analyzed the gene expression profile of a pure NC subpopulation isolated from *Sox10-IRES-Venus* mice and investigated whether these genes played a key role in the direct conversion of *Sox10-IRES-Venus* mouse embryonic fibroblasts (MEFs) into NC cells. The comparative molecular profiles of NC cells and neural tube cells in 9.5-day embryos revealed genes including transcription factors selectively expressed in developing trunk NC cells. Among 25 NC cell-specific transcription factor genes tested, SOX10 and SOX9 were capable of converting MEFs into SOX10-positive (SOX10+) cells. The SOX10+ cells were then shown to differentiate into neurons, glial cells, smooth muscle cells, adipocytes and osteoblasts. These SOX10+ cells also showed limited self-renewal ability, suggesting that SOX10 and SOX9 directly converted MEFs into NC cells. Conversely, the remaining transcription factors, including well-known NC cell specifiers, were unable to convert MEFs into SOX10+ NC cells. These results suggest that SOX10 and SOX9 are the key factors necessary for the direct conversion of MEFs into NC cells.

## Abstract (precis)

**Summary:** In this study, we identified the transcription factors specifically expressed in developing neural crest cells, and showed that SOX10 and SOX9 directly converted fibroblasts into neural crest cells.

## INTRODUCTION

Neural crest cells (NC cells) are migratory multipotent cells that give rise to diverse derivatives. During their movement or at target tissues, NC cells differentiate into many cell types, including neurons and glial cells of peripheral sensory and autonomic ganglia, Schwann cells, melanocytes, endocrine cells, smooth muscle, and skeletal and connective tissue cells of the craniofacial complex (Le Douarin and Kalcheim, 1999). Due to these characteristics of high migratory ability and multipotency, NC cells play critical roles in embryogenesis; and abnormalities in them have been shown to cause various serious diseases such as Waardenburg's syndrome, Hirschsprung's disease, CHARGE syndrome, and DiGeorge's syndrome (Hall, 2009).

A large number of earlier studies have approached the transcription factor/gene regulatory network for NC cell generation by using various species including zebrafish, *Xenopus*, chick, and mouse. The transcription factors/genes that control NC development are divided into subgroups based on their roles in each of the following stages of NC development: formation of the neural plate border (NBP), establishment of NC cell identity (the NC cell specification), and activation of the epithelial-mesenchymal transition (EMT) program (Simoes-Costa and Bronner, 2015). The first step of NC development is specification of the NBP. The NBP develops at the edge of the neural plate and contains the prospective NC cell population (Meulemans and Bronner-Fraser, 2002; Sauka-Spengler and Bronner-Fraser, 2008). The expression of a set of NBP specifier genes (e.g. *Msx*, *Pax3/7*, *Zic1*, *Dlx3/5*, *Tfap2a*) in the edge of the neural plate lead to the formation of the NBP (Khudyakov and Bronner-Fraser, 2009). The following step, the NC cell specification, is initiated by activation of transcription factors named NC cell specifiers, such as *Snai1/2*, *Tfap2a*, *Foxd3*, *Twist*, *Id*, *Myc*, and *Sox9/10* by NBP specifiers (Khudyakov and Bronner-Fraser, 2009; Simoes-Costa and Bronner, 2013). The expression of these NC cell specifiers establishes NC cell identity and activates the succeeding EMT program, which allows the NC cells to delaminate from the ectoderm. A combination of the NC cell specifiers is thought to maintain the NC cell in an undifferentiated state (Sauka-Spengler and Bronner-Fraser, 2008). The process of EMT involves the dissociation of intercellular connections, allowing the segregation of the NC cells into individual cells. NC cell specifier genes such as *Snai1/2*, *Tfap2a*, *Foxd3*, *Twist*, and *Sox10* participating in EMT are known to regulate the cell-surface molecules, resulting in NC cell delamination (Simoes-Costa and Bronner, 2015). Transcription factors/genes that control NC generation have thus been uncovered step by step; however, the pivotal factors responsible for NC identity yet remain to be elucidated.

The investigation of NC cells is hampered by difficulties in isolating and manipulating these cells. The NC cells emerge as a continuous cell population, progressively disperse, and invade neighboring tissues, thus making it difficult to separate and isolate them. *In vitro* assays have largely been limited to short-term primary cultures, because NC cells easily differentiate in culture. SOX10 is one of the NC cell specifiers and its expression starts in premigratory NC cells and continues in the migrating NC cells (Mollaaghababa and Pavan, 2003). Therefore, *Sox10* or its regulatory elements have been utilized as a reporter gene for NC cell. Transgenic mice in which the complete open reading frame of *Sox10* was replaced by lacZ sequences precisely marked the NC cells (Britsch et al., 2001). A transgenic mouse line with Sox10 distal enhancer MCS4 directing Cre expression was shown to be capable of inducing LacZ activity in NC cells when crossed with R26R:LacZ reporter mice (Stine et al., 2009). In other studies, a transgenic mouse line containing a bacterial artificial chromosome (BAC) in which tamoxifen-inducible Cre recombinase was inserted into the *Sox10* allele (Simon et al., 2012) or BAC with the *Sox10* allele replaced with *Venus* was used to establish transgenic mice (Shibata et al., 2010). These genetically engineered animals are favorable for the study of the NC cell. However, the expression of the reporter genes differs between transgenic strains, because the expression depends largely on the loci where the genes were inserted; and there exists a latency after Cre expression till the reporter gene is expressed. Some reports stated that Cre expression in response to the promoter/enhancer sequences of the marker genes did not faithfully recapitulate their endogenous expression (Ding et al., 2012; Ono et al., 2014).

We previously generated *Sox10-IRES-Venus* mice designed to express the green fluorescent protein ‘VENUS’ under the control of *Sox10*. The *Sox10-IRES-Venus* mice were inserted the *IRES-Venus* sequence after the termination codon of the *Sox10* genome sequence. By this knock-in strategy, the VENUS protein was faithfully expressed so as to allow tracing of the endogenous SOX10 expression, unlike the case for the other transgenic strategy. Therefore, the VENUS-marked cells in *Sox10-IRES-Venus* mouse embryos accurately reflect the behavior of the normal NC cells, and we are able to obtain purified NC cells from these embryos (Motohashi et al., 2014, 2011).

In this present study, we analyzed the gene expression profile of trunk NC cells in close comparison with that of neural tube cells and identified transcription factors that were specifically enhanced in trunk NC cells. The use of *Sox10-IRES-Venus* embryos enabled us to purify migrating NC cells from the embryo. We then tested transcription factors enhanced in these trunk NC cells for their capacity to directly convert mouse embryonic fibroblasts (MEFs) into NC cells. We investigated the cellular characteristics of these reprogrammed NC cells and discussed possible roles of the identified factors responsible for the direct conversion of MEFs into NC cells.

## RESULTS

### Identification of genes specifically expressed during early NC cell development

**Fig. 1.**
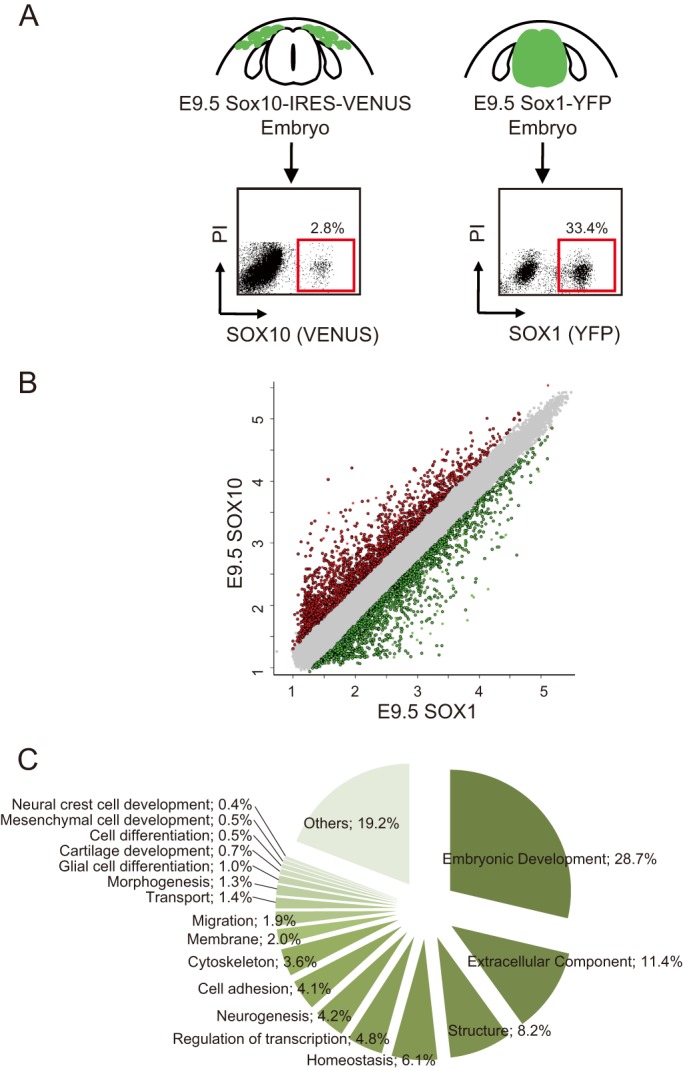
**Selection of genes that NC cells specifically expressed.** (A) Isolation of fluorescein-positive cells from the trunk regions of *Sox10-IRES-Venus* embryos and *Sox1-Cre/+;Rosa26R-YFP/+* embryos. Representative flow cytometry plots and the average percentages of SOX10+ cells and SOX1+ cells (gate outlined in red) are shown. PI, propidium iodide. (B) Scatter-plots comparing expression profiles between SOX10+ cells and SOX1+ cells. Red spots represent genes that showed higher than 2-fold expression levels and green spots represent those that showed lower than 2-fold expression levels in SOX10+ cells than in SOX1+ cells with significance of FDR<0.05. (C) Relative abundance of transcripts grouped by their molecular function. Genes that showed higher than 3-fold expression levels in SOX10+ cells than in SOX1+ cells were annotated with the ‘molecular function’ in the GO term.

To identify genes selectively expressed in NC cells, we isolated NC cells from the embryos of *Sox10-IRES-Venus* mice (Motohashi et al., 2011). The expression of SOX10 starts in premigratory NC cells and continues in migrating NC cells and their derivatives such as glial cells and melanocytes (Mollaaghababa and Pavan, 2003). The VENUS-marked cells in *Sox10-IRES-Venus* mouse embryos have been shown to accurately reflect the behavior of normal NC cells (Motohashi et al., 2014, 2011). We collected embryonic day (E)9.5 *Sox10-IRES-Venus* embryos from one pregnant *Sox10-IRES-Venus* female and then isolated SOX10-positive (SOX10+) NC cells from the trunk region of these embryos. Transverse sections of E9.5 *Sox10-IRES-Venus* embryos showed the presence of SOX10+ cells close to the dorsal side of the neural tube, corresponding well to the NC cells of this embryonic stage (Fig. S1). Then we isolated RNA samples from these SOX10+ cells and used them for gene-array analysis (Fig. 1A). Trunk neural tube cells from which trunk NC cells were derived were suitable for a closer control of trunk NC-specific gene expression. We also employed *Sox1-Cre/+;Rosa26R-YFP/+* mice expressing YFP under the control of *Sox1*, which allowed YFP to be expressed in developing neural tissues including the neural tube (Srinivas et al., 2001; Takashima et al., 2007) for the gene-array analysis. Almost all neural tube cells express YFP in *Sox1-Cre/+;Rosa26R-YFP/+* mouse embryos at E9.5 (Takashima et al., 2007). We isolated SOX1-positive (SOX1+) cells from the trunk region of E9.5 embryos and used them as a reference in the gene-array analysis (Fig. 1A). Table S1 shows a list of the gene array, and raw gene array data are available at Gene Expression Omnibus, http://www.ncbi.nlm.nih.gov/geo/query/acc.cgi?token=wpqnmeykbrcbfoh&acc=GSE63896, under accession number GSE63896.

The mean log-intensity chart for E9.5 SOX10+ cells vs E9.5 SOX1+ cells showed that 1932 genes were significantly overexpressed in the E9.5 SOX10+ cells (Fig. 1B). Among the genes identified, genes that showed higher than 3-fold expression levels in SOX10+ cells than in SOX1+ cells were selected and were annotated with the ‘molecular function’ in the GO term. The results are listed in Table S2, and Fig. 1C shows the relative abundance of transcripts grouped by their molecular function. Genes related to embryonic development accounted for 28.7% of the up-regulated transcripts, followed by extracellular components (11.4%), structure (8.2%), and homeostasis (6.1%). Genes related to mesenchymal cell development and NC cell development in the GO term accounted for 0.5% and 0.4%, respectively. The up-regulated genes in SOX10+ cells included those for Wnt signals (*Wnt2*, *Wnt6*), transcription factors known to generate NC cells (*Dlx*s, *Snail1*, *FoxD3*, *Sox9*, *Sox10*; Simoes-Costa and Bronner, 2015), genes for NC cell migration; *Cxcl12* (Belmadani et al., 2005), genes used as NC cell markers (*P0*, *Ngfr*; Lemke et al., 1988; Stemple and Anderson, 1992), and genes used as mesenchymal cell markers (*Pdgfrα*, *Pdgfrβ*; Andrae et al., 2008). Cadherin family members that play critical roles in the epithelial-mesenchymal transition (EMT) and in NC cell generation were also significantly expressed (*Chd6*, *Cdh11*, *Cdh19*; Park and Gumbiner, 2010; Takahashi and Osumi, 2005; Vallin et al., 1998). Genes for normal development of the enteric nerves, which are derived from NC cells (*Gfra1*, *Ednrβ*; Baynash et al., 1994; Cacalano et al., 1998), and genes known to play roles in glial cell differentiation (*Dhh*, *Plp*) were also among the up-regulated genes (Bitgood and McMahon, 1995; Griffiths et al., 1998). The expression of *Hoxa3*, which is localized to NC cells in the anterior part of the pharyngeal arch (Manley and Capecchi, 1995), was not significantly increased, suggesting that the array data reflected the expression profile of the trunk NC cells.

### Direct conversion of fibroblasts into SOX10+ NC cells by the NC cell-specific transcription factors

Direct reprogramming techniques have recently been reported to convert dermal fibroblasts into cells of specific cell lineages such as neural stem cells (Han et al., 2012; Thier et al., 2012), hepatocytes (Huang et al., 2011; Sekiya and Suzuki, 2011), and cardiac cells (Ieda et al., 2010). We hypothesized that the genes specifically expressed in the SOX10+ NC cells might have the potential to convert the identity of the target cells to that of NC cells. For this purpose, we selected the transcription factor genes included in the ‘transcription factor activity’, ‘transcription factor complex’, ‘neural crest cell development’, and ‘mesenchymal cell development’ categories among the ‘GO molecular function term’. Furthermore, we narrowed the candidate transcription factors by using their NC cell-like *in situ* expression patterns among the openly available *in situ* gene expression patterns (EMBRYS: http://embrys.jp/embrys/html/MainMenu.html) (Yokoyama et al., 2009). Thus we selected the 25 transcription factor genes listed in Table 1.

**Table 1.**
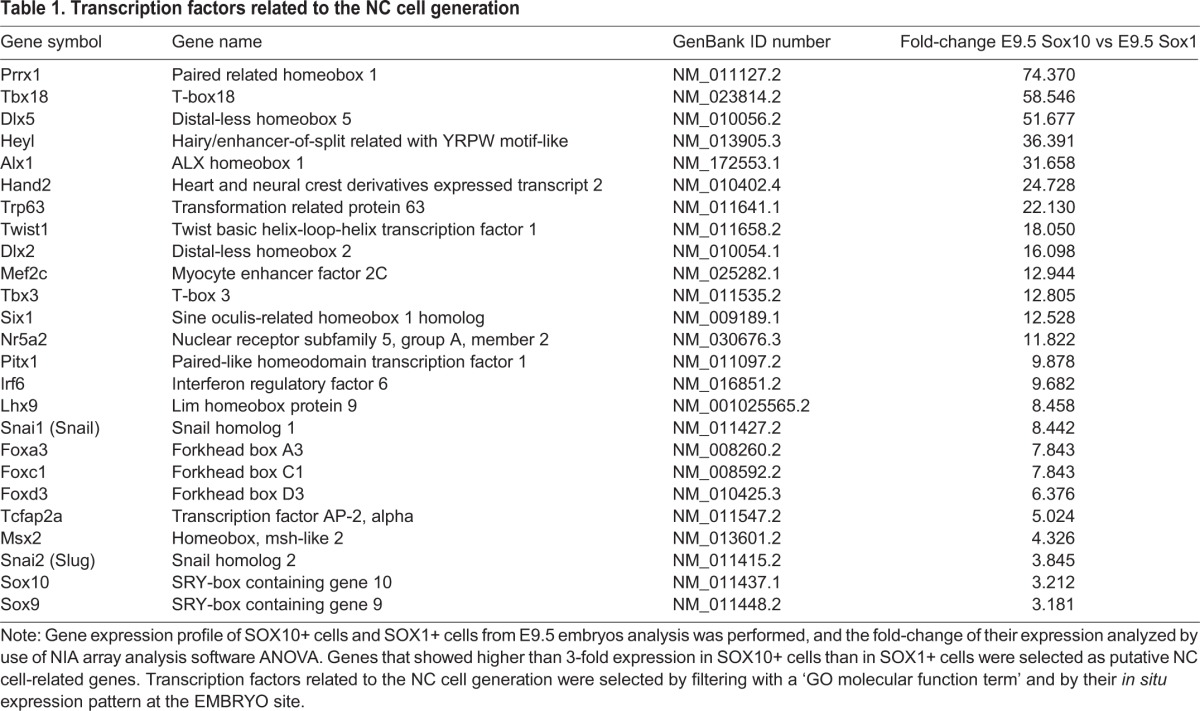
**Transcription factors related to the NC cell generation**

**Fig. 2.**
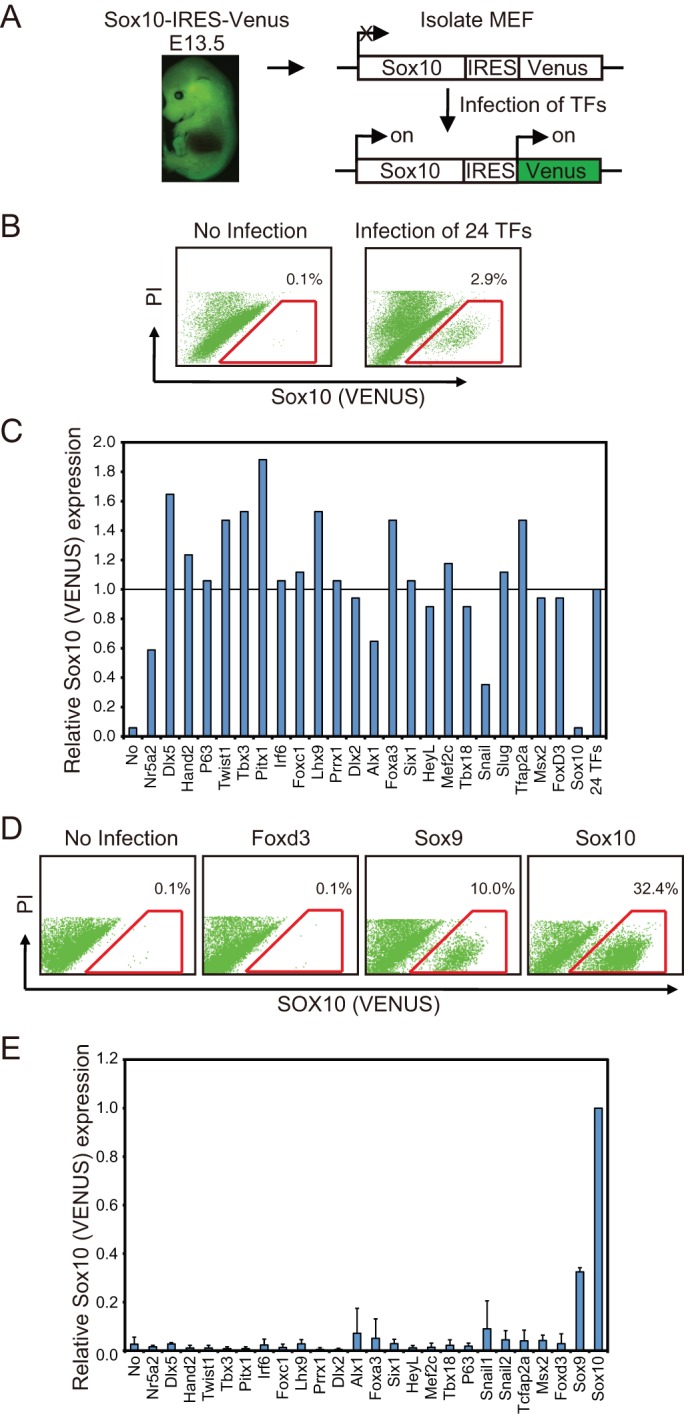
**SOX9 or SOX10 convert MEFs into SOX10+ cells.** (A) Experimental scheme for the transcription factor-mediated conversion of *Sox10-IRES-Venus* MEFs into SOX10+ cells. (B) Representative flow cytometry plots of *Sox10-IRES-Venus* MEF with and without infection with 24 transcription factors (24 TFs). The numbers show the average percentage of SOX10+ cells (red line gate). PI, propidium iodide. (C) Identification of the transcription factors directly involved in this conversion. Transcription factors, except for the indicated transcription factor, were expressed in *Sox10-IRES-Venus* MEF, and the rate of generation of SOX10+ cells was analyzed by flow cytometry. The longitudinal axis indicates the relative ratio of the percentage of SOX10+ cells in 24 TF-infected fibroblasts. Experiments were performed two times. (D) Representative flow cytometry plots of *Sox10-IRES-Venus* MEF infected with *Foxd3*, *Sox9* or *Sox10*. (E) The indicated transcription factor was expressed in *Sox10-IRES-Venus* MEF, and the rate of generation of SOX10+ cells was analyzed. The longitudinal axis indicates the relative ratio of the percentage of SOX10+ cells in *Sox10*-infected fibroblasts. The experiments were performed three times. Error bars represent s.d.

MEFs generated from *Sox10-IRES-Venus* mice embryos were used as the cells to be directly converted into NC cells because the MEFs started to express VENUS fluorescent protein once their NC identity had been acquired (Fig. 2A). Since *Sox9* and *Sox10* belong to the SoxE family and both are known to control NC generation (Cheung and Briscoe, 2003; Hong and Saint-Jeannet, 2005; Kim et al., 2003), we excluded *Sox9* from the first experiment. So 24 candidate transcription factors were selected from the expression clones in the human proteome expression resource (HuPEX) library (HuPEX clones in HGPD, http://www.HGPD.jp/) and each of them was cloned into a retrovirus vector system. The selected transcription factors all showed 82-100% amino acid sequence homologies with those of the mouse. The MEFs infected with all 24 candidate genes were cultured with the culture-supernatant of ST2 cells, as the ST2 cell line was previously reported to allow the induction of NC-like cells from mouse ES cells (Motohashi et al., 2007) and to maintain undifferentiated NC cells (Motohashi et al., 2011). After 11 days of cultivation, we observed SOX10+ cells among the infected *Sox10-IRES-Venus* MEFs (Fig. 2B). To identify the transcription factors directly involved in the conversion, we removed one gene from the set of 24 genes and overexpressed the remaining 23 genes in MEFs. As shown in Fig. 2C, only the gene set lacking *Sox10* showed markedly reduced generation of SOX10+ cells, suggesting that *Sox10* was solely responsible for the generation of SOX10+ cells. When each gene was singly overexpressed in MEFs, SOX10+ cells were generated only from the *Sox10-*infected MEFs (Fig. 2D,E). When *Sox9* was singly overexpressed in MEFs, SOX10+ cells were also generated (Fig. 2D,E). Other transcription factors well known to be NC cell specifiers, such as *Foxd3*, *Tcfap2a* or EMT-related genes *Snail1* and *Snail2,* scarcely generated SOX10+ cells from the MEFs (Fig. 2D,E).

**Fig. 3.**
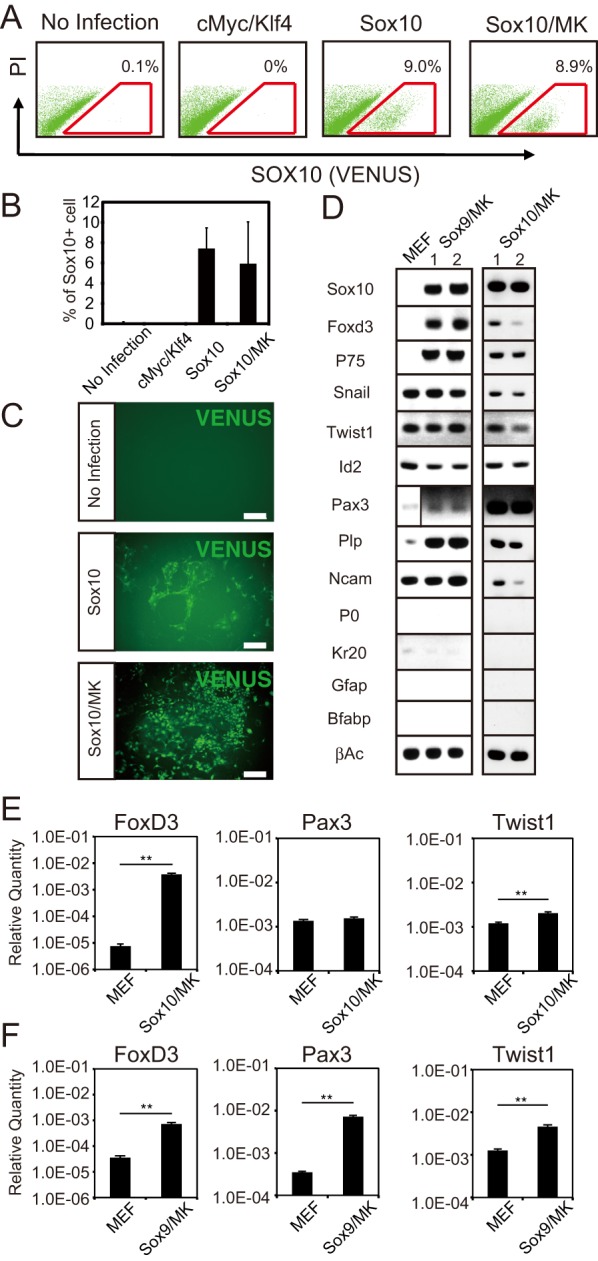
**SOX10/c-MYC/KLF4 converted MEF into SOX10+ NC cells.** (A) Representative flow cytometry plots of *Sox10-IRES-Venus* MEFs transfected with *c-Myc*/*Klf4*, *Sox10* or *Sox10*/*c-Myc*/*Klf4* (Sox10/MK). The numbers show the average percentage of SOX10+ cells (gate outlined in red). PI, propidium iodide. (B) The graph shows the average percentage of SOX10+ cells obtained with each transgene. (C) Representative photomicrographs of SOX10 (VENUS)+cells generated from the expression of *Sox10* and that of *Sox10/MK*. Scale bar=200 µm. (D) RT-PCR analysis of the SOX10+ cells generated from Sox9/MK- and Sox10/MK-infected MEFs. The number shows the SOX10+ cells generated in different experiments. (E,F) qPCR analysis of NC cell marker genes in MEFs and the SOX10+ cells generated from Sox9/MK- and Sox10/MK-infected MEFs. The graph depicts the levels of *Foxd3*, *Pax3* and *Twist1*. The experiment was performed twice and the expression levels relative to the β-actin expression are shown. Error bars represent s.d.; **P<0.01.

We then cultured the isolated SOX10+ cells from *Sox10-*infected MEFs under the previously reported NC cell culture conditions (Morrison et al., 1999; Stemple and Anderson, 1992). While the isolated SOX10+ cells adhered to the culture dishes after one day of cultivation, these cells did not proliferate under these culture conditions; and most of the cells detached from the cultured dishes after approximately six days of cultivation. SOX family transcription factors have recently been used for the direct reprogramming of fibroblasts in combination with KLF4 and c-MYC (Hiramatsu et al., 2011; Thier et al., 2012). c-MYC is a well-known stimulator of cell-cycle progression (Thompson et al., 1985) and plays a role in the formation of NC precursor cells (Bellmeyer et al., 2003). KLF4 is known to be involved in diverse biological processes including proliferation and apoptosis (Black et al., 2001; Ghaleb et al., 2005; McConnell et al., 2007), and these two genes also constitute Yamanaka factors. We co-expressed c-MYC and KLF4 with SOX10 and observed no significant difference in the rate of SOX10+ cell generation when compared with that obtained when SOX10 was solely expressed (Fig. 3A,B). The SOX10+ cells isolated from MEFs co-expressing SOX10/c-MYC/KLF4 were maintained and markedly proliferated without detachment from culture dishes under the NC cell culture conditions used (Fig. 3C).

A chronological flow cytometric analysis of MEFs co-infected with *Sox10*/*c-Myc*/*Klf4* revealed that SOX10+ cells began to emerge after six days of cultivation and increased in number in a time-dependent manner (Fig. S2). Since SOX10 is known as a marker for glial cells (Mollaaghababa and Pavan, 2003), it is possible that the SOX10+ cell population that had emerged after six days of cultivation contained fully differentiated glial cells. We examined the chronological expression of NC cell and glial cell markers in the generated SOX10+ cells by using reverse transcription PCR (RT-PCR). The NC marker *P75* began to be expressed, and the expression of *Foxd3* was significantly increased on day 6 of cultivation; whereas the glial cell marker *Gfap* was expressed after six days of cultivation, and its expression significantly increased in a time-dependent manner (Fig. S3). These results indicated that the MEFs induced to express SOX10/c-MYC/KLF4 were converted to NC cells after six days of cultivation and that these cells then gradually differentiated into glial cells. We isolated SOX10+ cells after six days of cultivation and then cultured them for another six days under the NC cell culture conditions. These cultured SOX10+ cells were found to maintain the expression of the NC cell marker genes *Foxd3*, *P75*, *Snail1*, *Twist1*, *Id2*, and *Pax3*, while losing the expression of the glial cell markers *Gfap*, *Krox20*, and *P0* (Fig. 3D). The SOX10+ cells isolated from MEFs co-expressing SOX9/c-MYC/KLF4 also continued to express *Foxd3*, *P75*, *Snail1*, *Twist1*, *Id2*, and *Pax3* under the NC cell culture conditions (Fig. 3D).

Next, we measured the mRNA level of the *Foxd3*, *Pax3*, and *Twist1* of the SOX10+ cells after six days under the NC cell culture conditions by quantitative PCR (qPCR). In the generated SOX10+ cells, the mRNA levels of *Foxd3* and *Twist1* were higher than those in MEFs (Fig. 3E,F). There was a significant difference in the mRNA level of *Pax3* between the SOX10+ cells generated from SOX9/c-MYC/KLF4-expressed MEFs and control MEFs (Fig. 3F). However, there was no statistical difference in the *Pax3* expression between the SOX10+ cells generated from SOX10/c-MYC/KLF4-expressed MEFs and control MEFs (Fig. 3E). This finding suggests that SOX10 and SOX9 directly converted MEFs into NC cells after six days of cultivation with the supernatant of ST2 cell cultures.

To confirm that MEFs were directly converted into NC cells, we generated MEFs from *P0*-promoter *Cre/loxP-LacZ;* *Sox10-IRES-Venus* transgenic mouse embryos and overexpressed SOX10/c-MYC/KLF4 in these MEFs (Fig. S4A). P0 was shown to be transiently activated in migrating NC cells in early chick embryos (Lemke et al., 1988); and so the *P0*-promoter *Cre/loxP-LacZ* was used as a NC cell-specific marking system. The MEFs overexpressing SOX10/c-MYC/KLF4 generated SOX10+ cells after six days of cultivation in the culture-supernatant of ST2 cells (Fig. S4B). The isolated SOX10+ cells were markedly positive for LacZ activity as compared with the uninfected MEFs (Fig. S4C). The percentage of LacZ activity-positive cells among the isolated SOX10+ cells was 39.0±2.8%, and that among the uninfected MEFs, 7.9±0.8% (Fig. S4D). We further analyzed the expression of the NC cell marker P75 in the generated SOX10+ cells. Approximately 40% of the generated SOX10+ cells expressed P75 (Fig. S4E). These results indicate that the generated SOX10+ cell population contained NC-lineage cells.

The MEFs that we established from *Sox10-IRES-Venus* embryos contained a trace amount of SOX10+ cells (Fig. 3A); so, therefore, the SOX10+ cells generated might not have been derived from SOX10-negative (SOX10−) MEFs, but simply expanded from these contaminating SOX10+ cells. To eliminate this possibility, we sorted SOX10-negative (SOX10−) cells at 1 cell/well into wells of a 96-well dish after two days of the induction of *Sox10/c-Myc/Klf4*. The SOX10− cells were cultured for six days and then analyzed for the expression of SOX10. (Fig. S5A). Approximately 13.1-46.1% of the SOX10− cells expressed SOX10 after six days of cultivation (Fig. S5B,C), suggesting that the SOX10+ cells had truly originated from SOX10− MEFs.

### Directly converted SOX10+ NC cells differentiate into neural cells, glial cells, smooth muscle cells, osteocytes, and adipocytes

**Fig. 4.**
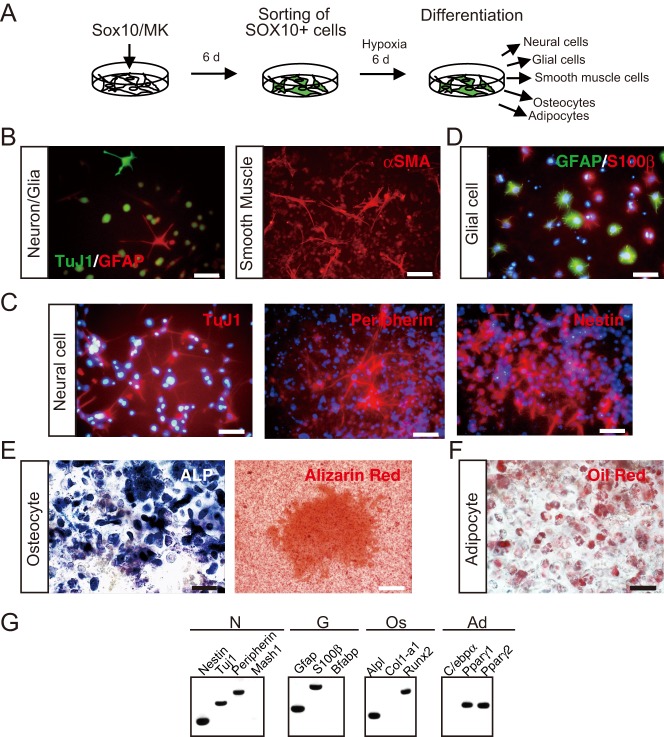
**Converted SOX10+ NC cells differentiated into neural cells, glial cells, osteocytes and adipocytes.** (A) Experimental scheme for the differentiation of SOX10+ NC cells converted from MEFs. Converted SOX10+ NC cells were cultured under NC cell culture conditions with hypoxia. After expansion for six days, the converted SOX10+ NC cells were cultured in each type of differentiation medium. (B-F) Immunostaining image of the differentiated SOX10+ NC cells. TuJ1+ neural cells, GFAP+ glial cells, and α smooth muscle actin (αSMA)+ smooth muscle cells were differentiated from isolated SOX10+ NC cells in the NC cell differentiation medium (B). In specific differentiation medium, the converted SOX10+ NC cells differentiated into TuJ1+, peripherin+, and nestin+ neural cells (C); GFAP+ and S100β+ glial cells (D); osteocytes with alkaline phosphatase activity (ALP) and positive staining with Alizarin Red (E); and adipocytes with positive Oil Red O staining (F). Scale bar=50 µm in B left, C,D,E left, F; 100 µm in B right, E right. Nuclei were stained with Hoechst 33258 (Blue). (G) RT-PCR analysis of neural cells, glial cells, osteocytes and adipocytes differentiated from Sox10+ NC cells. N, neural cells differentiated from Sox10+ NC cells; G, glial cells differentiated from Sox10+ NC cells; Os, osteocytes differentiated from Sox10+ NC cells; Ad, adipocytes differentiated from Sox10+ NC cells.

Next, we evaluated the differentiation potencies of the converted SOX10+ NC cells (Fig. 4A). After six days of cultivation under NC cell culture conditions, the media were changed to the previously reported NC cell differentiation medium (Morrison et al., 1999). Clusters or colonies including class III β-tubulin (TuJ1)-positive neural cells, GFAP-positive glial cells, and α smooth muscle actin (αSMA)-positive smooth muscle cells emerged after eight days of cultivation (Fig. 4B). This result demonstrated that the converted SOX10+ NC cells possessed the same differentiation potency as NC cells. To cause the cells to effectively differentiate into NC derivatives, we changed the medium to differentiation medium specific for neural cells, glial cells, osteocytes, and adipocytes after expansion for six days. After 21 days of cultivation in the differentiation medium specific for neural cells, the results of an immunostaining analysis revealed that isolated SOX10+ NC cells differentiated into TuJ1+ neural cells (Fig. 4C). These neural cells also expressed other neural cell markers peripherin and nestin (Fig. 4C). RT-PCR showed that the differentiated neural cells expressed *β-tubulin* (Tuj1), *Nestin*, and *Peripherin* (Fig. 4G), but not the autonomic neural marker *Mash-1* (Fig. 4G). GFAP+ glial cells also differentiated from the isolated SOX10+ NC cells after 21 days of cultivation in the differentiation medium specific for glial cells (Fig. 4D). Some of these differentiated GFAP+ glial cells also expressed the mature glial cell marker S100β, whereas most of the glial cells expressed S100β or GFAP (Fig. 4D). RT-PCR showed that the differentiated glial cells expressed *S100β* or *Gfap*, but not another mature glial cell marker, *B-fabp* (Fig. 4G). Osteocytes were observed as alkaline phosphatase activity-positive cells and alizarin red-stained cells (Fig. 4E), and adipocytes were observed Oil Red O-positive cells (Fig. 4F), after 21 days of cultivation. The differentiated osteocytes expressed *Alpl* (alkaline phosphatase) and *Runx2*, but not *Col1a1* (type I collagen; Fig. 4G); and the adipocytes expressed *Pparγ1* and *Pparγ2*, but not *C/ebpα* (Fig. 4G). The SOX10+ NC cells generated from *Sox9*/*c-Myc*/*Klf4*-transfected MEFs were also capable of differentiating into TuJ1-positive neural cells, GFAP-positive glial cells, alkaline phosphatase activity-positive osteocytes, and oil red O-positive adipocytes after 21 days of cultivation (Fig. S6A-D).

In order to make it clear that the neural cells and glial cells had differentiated from the isolated SOX10+ cells, we chronologically observed 2 clusters generated from the isolated SOX10+ cells. These clusters were generated from the SOX10+ cells under the NC cell culture conditions after six days of culture, and they gradually expanded in NC cell differentiation medium (Fig. S7A). After 16 days of cultivation, one cluster generated glial cells and neural cells (Fig. S7B); and the other, glial cells (Fig. S7C). This result showed that the generated neural cells and glial cells had been derived from the isolated SOX10+ cells.

Multipotent cells existing among the fibroblasts may have differentiated into NC cell derivatives (Fernandes et al., 2004; Wong et al., 2006). To eliminate this possibility, we cultured MEFs under NC cell culture conditions for six days (Fig. S8A). Most MEFs detached from the culture dishes after three or four days of cultivation (Fig. S8B), and even subtly adhering MEFs never differentiated into neural cells or glial cells in the differentiation medium specific for glial cells or neural cells (Fig. S8C,D).

Furthermore, we cultured MEFs infected with *c-Myc* and *Klf4* under NC cell culture conditions (Fig. S9A). The infected MEFs were maintained in the medium for six days, after which the medium was changed to differentiation medium specific for neural cells, glial cells, osteocytes, and adipocytes. After 21 days of culture, no neural cells, no glial cells and no osteocytes had differentiated from the *c-Myc* and *Klf4* infected MEFs (Fig. S9B-D). On the contrary, some Oil Red O-positive adipocytes were observed in the cultures of the infected MEFs (Fig. S9E). MEFs are known to be a heterogeneous population including pre- or pro-adipocytes (Driskell et al., 2013). The starting MEFs robustly expressed *Pparγ1* and *Paprγ2*, showing that the MEFs contained pre- or pro-adipocytes (Fig. S9F). On the contrary, neither *Pparγ1* nor *Pparγ2* was expressed in the SOX10+ cells generated by *Sox10*/*c-Myc*/*Klf4* infection, showing that the SOX10+ cell population did not contain pre- or pro-adipocytes (Fig. S9F). This finding strongly suggests that the adipocytes generated by cultivation of the converted SOX10+ cells had differentiated from the SOX10+ cells, not from contaminating pre- or pro-adipocytes. These results thus demonstrated that the converted SOX10+ NC cells possessed the ability to differentiate into neural cells, glial cells, smooth muscle cells, adipocytes, and osteocytes, similar to *in vivo* NC cells.

### Self-renewal capacity of converted SOX10+ NC cells

**Fig. 5.**
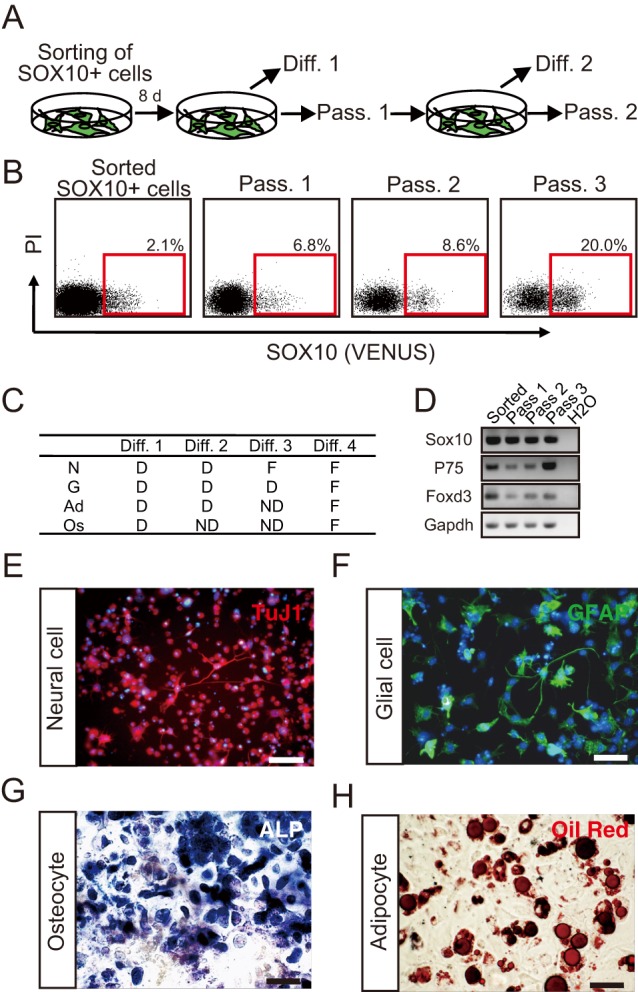
**Self-renewal of converted SOX10+ NC cells.** (A-B) Experimental scheme for the self-renewal of SOX10+ NC cells converted from MEFs. Converted SOX10+ NC cells were isolated and cultured under NC cell culture conditions. After eight days, some generated colonies were passaged (Pass. 1), while others were differentiated into NC derivatives (Diff. 1). The passages were serially performed (A). In each passage, the rate of generation of SOX10+ cells was analyzed by flow cytometry (B). (C) Differentiation abilities of the converted SOX10+ NC cells after serial passages. ‘D’ indicates that differentiated cells were detected; ‘F’, that a few differentiated cells were detected; and ‘ND’, that no differentiated cells were detected. N, neural cells; G, glial cells; Ad, adipocytes; Os, osteocytes. (D) RT-PCR analysis of NC cell markers in serially passaged SOX10+ NC cells. (E-H) Immunostaining image of differentiated SOX10+ NC cells at Diff. 4. SOX10+ cells differentiated into neural cells (E), glial cells (F), osteocytes (G), and adipocytes (H). Nuclei were stained with Hoechst 33258 (Blue). Scale bar=50 µm.

We dissociated clusters or colonies generated from the isolated SOX10+ NC cells after six days of expansion under NC cell culture conditions, counted the number of SOX10+ cells, and re-cultured the dissociated cells under NC cell culture conditions (Fig. 5A). These NC cell culture conditions were previously shown to maintain the self-renewal capacity of *in vivo* NC cells after serial passages (Morrison et al., 1999). As shown in Fig. 5B, the converted SOX10+ NC cells could propagate under the NC culture conditions during serial passage. To evaluate the differentiation ability after passages, we cultured some clusters or colonies in the differentiation medium to generate neural cells, glial cells, osteocytes, and adipocytes. Although osteogenesis and adipogenesis were detected in the first culture, these cells were gradually lost after subsequent passage (Fig. 5C,G,H). Even though neural cells and glial cells were still generated after the serial passage, their numbers gradually decreased (Fig. 5C,E,F). The NC cell markers *P75* and *Foxd3* were expressed even after serial passages (Fig. 5D). We analyzed the expression of the transgenes *Sox10*, *c-Myc* and *Klf4* after serial passages and observed that all transgenes were expressed even after the third passage (Fig. S10). The SOX10+ NC cells converted from *Sox9*/*c-Myc*/*Klf4*-transfected MEFs showed self-renewal; however, they lost their ability for osteogenesis after the first passage (Fig. S6E-G). These results suggest that converted SOX10+ NC cells maintained a limited, but significant self-renewal capacity, under NC cell culture conditions after serial passages.

### SOX10+ NC cells show extended motility upon transplantation *in ovo*

**Fig. 6.**
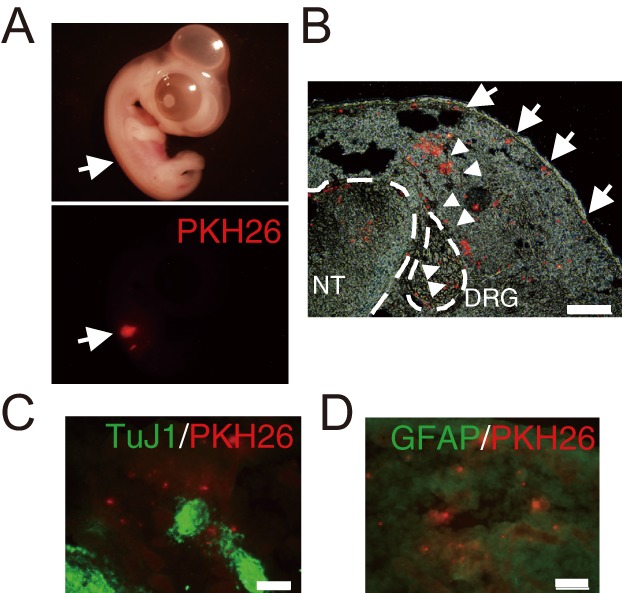
**Converted SOX10+ NC cells migrated along NC cell migration pathways.** (A) HH stage 29-30 chick embryo transplanted PKH26-labeled SOX10+ NC cells. Arrows indicate the transplanted SOX10+ NC cells. (B) Cross section of a HH stage 29-30 chick embryo. PKH26-labeled SOX10+ NC cells migrated along the ventral pathway (arrowheads) and dorsolateral pathway (arrows). DRG, dorsal root ganglion; NT, neural tube. Scale bar=200 µm. (C-D) Immunostaining of cross sections with TuJ1 (neural cells; C) and anti-GFAP (glial cells; D). Scale bar=50 µm.

To determine whether SOX10+ NC cells exhibited migration potency similar to that of NC cells *in vivo*, we transplanted the isolated and expanded SOX10+ NC cells into the chick NC cell migratory stream *in ovo* at Hamburger and Hamilton stage 17-18. After three days (stage 30), the transplanted cells had remained in the embryo (Fig. 6A). Although the transplanted uninfected MEFs did not exhibit migration capacity (Fig. S11A,B), some transplanted SOX10+ cells migrated along the NC cell migration pathways, the dorsolateral pathway (Fig. 6B, arrow) and the ventral one (Fig. 6B, arrowhead). Some transplanted cells arrived in the dorsal root ganglion (Fig. 6B, arrowhead). However, immunostaining showed that these transplanted cells did not differentiate into TuJ1+ neural cells or GFAP+ glial cells (Fig. 6C,D). Thus, these transplanted SOX10+ NC cells possessed migration ability similar to that of *in vivo* NC cells.

## DISCUSSION

In this study, we identified genes strongly enriched in migrating SOX10+ trunk NC cells purified from *Sox10-IRES-Venus* embryos. Previously, genes with enhanced expression in cranial NC cells were studied by using chick embryos transfected with a reporter construct containing a Sox10E2 enhancer sequence (Simoes-Costa et al., 2014); and many enzymes, kinases, and transcription factors including well-known NC cell specifiers and NBP specifiers were identified. Our study also showed that NC cell specifiers *Foxd3*, *Sox10*, and *Snail2*, as well as the NBP specifier, *Tcfap2*, were markedly expressed in mouse trunk NC cells. Furthermore, other well-known NC cell specifiers or NBP specifiers such as *Sox9*, *Msx*s and *Dlx*s were significantly expressed in the trunk NC cells. Conversely, *Pax3* and *Zic1* were not detected by our array data analysis. *Pax3* and *Zic1* cooperate as NBP specifiers and directly activate NC specifiers during NC development (Milet et al., 2013). *Pax3* and *Zic1* could not be detected in our comparative molecular analysis of NC cell and neural tube cell profiles because of their markedly high expression in the neural tube (Mansouri et al., 2001; Nagai et al., 1997). Another NBP specifier gene, *Dlx3*, was identified as a gene having 2.3 times higher expression in NC cells (Table S1), indicating that the SOX10+ cell population contained the generating NC cells. On the other hand, the absence of *Pax3* and *Zic1* in our analysis possibly suggested that the isolated SOX10+ cell population contained NC cells at the late stage of migration (Milet et al., 2013). It also should be noted that the expression of *Mitf* and *Mcam,* which are genes for melanocyte generation (Carreira et al., 2006; Nagai et al., 1997; Sers et al., 1993) were detected in our analysis. Furthermore, the glial cell related genes expression was detected in the isolated SOX10+ cells (Fig. 1C). It is possible that the isolated SOX10+ cells were NC cells at later stages; i.e. a mixture of the forming and migrating NC cells and some cells started to express differentiated markers for NC cell descendants. The above-mentioned studies also showed that chick cranial NC cells express genes related to chondrocyte development, such as *Collagen 2*, *9*, and *11* (Simoes-Costa et al., 2014); however, we did not observe such expression in our analysis. As only cranial NC cells are known to have chondrogenic potential (Graham et al., 2004), the absence of chondrocyte markers accurately showed that the isolated SOX10+ cells were the trunk NC cells. *Rhox*s, which are homeobox genes, were also enhanced in expression in mouse trunk NC cells. The *Rhox* family is mainly expressed in the testis and ovary during embryonic development to control male and female reproduction (Maclean et al., 2005), and overexpression of *Rhox5* in mouse ES cells perturbs their differentiation (Fan et al., 1999).

Our identified transcription factors dominantly expressed in trunk NC cells were tested for their ability to directly convert MEFs into NC cells, and the results demonstrated that MEFs were directly converted into SOX10+ NC cells by the forced-expression of SOX9 or SOX10 alone. SOX9 and SOX10 belong to same Sox family subgroup ‘SoxE group’, and they are expressed and function in NC development (Hong and Saint-Jeannet, 2005). Recent analysis of the upstream of the *Sox10* gene showed that SOX10 directly binds to its own enhancer, U3, which is situated 28 kb upstream of the *Sox10* to regulate its own expression (Wahlbuhl et al., 2012). Thus, the forced-expression of SOX10 would be expected to start further induction of SOX10; and this positive-feedback expression of *Sox10* may have easily converted the MEFs into NC cells. SOX9 also binds to the U3 enhancer of *Sox10* and induces *Sox10* expression (Wahlbuhl et al., 2012). The forced expression of SOX9 likely to up-regulated *Sox10* in the MEFs and thus stimulated the conversion of MEFs into NC cells.

It should be noted that among the 25 trunk NC cell-specific transcription factor genes tested, *Sox10* and *Sox9* were the only effective genes for the conversion of the MEFs into NC cells. That is, other transcription factors known to play a role in EMT or NC cell specification, such as TCFAP2a, FOXD3, SNAIL1, and TWIST1 (Table 1), were not effective individually (Fig. 2C,D,E). PAX3, not picked up in our array analysis but known to be related to NC cell specification, was tested and found not to generate SOX10+ cells from the MEFs (data not shown). This finding probably show that SOX10 and SOX9 are the pivotal factors to direct NC cell identity. But the fibroblasts are mesenchymal lineage cells that already expresses SNAIL1, SNAIL2, and TWIST1 (Huleihel et al., 2014) and we also confirmed that *Twist1*, *Snail1* and *Snail2* were expressed in the *Sox10-IRES-Venus* MEFs (Fig. 3D, Fig. S3). The mesenchymal characteristics of fibroblasts is likely the reason that co-expression of *Twist*, *Snail1*, or *Snail2* genes was not necessary for *Sox10*-induced reprogramming. This hypothesis might well be tested by using other cell types such as epithelial cells or hematopoietic cells as a target of direct reprogramming. On the other hand, the SOX10+ cells generated by the expression of solely SOX10 or SOX9 were not able to be maintained in *in vitro* culture without the expression of KLF4/c-MYC and they gradually lost their differentiation potency after serial passages (Fig. 5C; Fig. S6). It is likely that SOX10 or SOX9 expression was not sufficient for maintaining the NC cell state, particularly for the self-renewing capability. The combinational expression of SOX10 or SOX9 with the other identified factors might permit the MEFs to fully acquire the NC cell property.

We co-expressed *Sox10* with *c-Myc* and *Klf4*, genes directly related to cell maintenance or cell propagation, in MEFs for the maintenance or proliferation of the converted NC cells. c-MYC is a well-known stimulator of the cell cycle (Thompson et al., 1985), and is known to be expressed in the neural plate border and to be required for the induction of NC cell precursors (Bellmeyer et al., 2003). KLF4 has been shown to have diverse regulatory functions in terms of proliferation, differentiation, and development (Black et al., 2001; Ghaleb et al., 2005; McConnell et al., 2007). The same strategy was adopted in a recent study using SOX family transcription factors together with KLF4 and c-MYC for the direct reprogramming of mouse fibroblasts into chondrocytes or neural stem cells (Hiramatsu et al., 2011; Thier et al., 2012). Furthermore, c-MYC and KLF4 were reported to facilitate the chromosome remolding process (Amati et al., 2001; Sridharan et al., 2009). Although the co-expression of c-MYC and KLF4 with SOX10 did not increase the number of SOX10+ cells (Fig. 3A), SOX10+ cells came to be stably maintained in culture.

A recent study reported that human fibroblasts can be converted into NC cells by the expression of SOX10 alone and that the converted NC cells are able to proliferate in culture after multiple passages (Kim et al., 2014). Although the fibroblasts used were a different species than the one we used in our experiment, the main difference between these findings and ours is the culture conditions of fibroblasts during their conversion into NC cells. Kim et al. converted human fibroblasts under culture conditions with strong epigenetic modifiers and WNT activator and BMP4. Activation of WNT and BMPs signals may be critical, because these factors have been shown to play roles in the *in vivo* induction of NC cells (Knecht and Bronner-Fraser, 2002). We used the supernatant of cultured ST2 cells for the conversion of MEFs, and these cells were previously reported to express *Wnt*s and *Bmp*s (Goseki-Sone et al., 2002; Ouji et al., 2012; Rawadi et al., 2003). We also found the expression of *Wnt3a*, *Bmp2,* and *Bmp4* in ST2 cells by RT-PCR analysis (data not shown). Now we think that WNT3a, BMP2, and BMP4 were likely the crucial factors secreted from ST2 cells for the conversion, but that these factors in the ST2 cell culture supernatant might not have been adequate to complete the direct conversion of MEFs.

We also found that the expression of transgenes *Sox10*, *c-Myc* and *Klf4* was sustained even after serial passages (Fig. S10). The silencing of *c-Myc* and *Klf4* is known to be required for iPSC generation (Takahashi and Yamanaka, 2006). Furthermore, transgene silencing has been identified as a prerequisite for the normal differentiation of iPSCs (Brambrink et al., 2008). The sustained expression of transgenes may add a negative impact on the differentiation potencies of converted SOX10+ NC cells. For example, although SOX10+ NC cells were maintained under NC culture conditions during the serial passages, their differentiation potency gradually decreased; and they did not exhibit the capacity for osteogenesis or adipogenesis after serial passaging (Fig. 5C). Furthermore, SOX10+ NC cells converted from MEFs did not differentiate into NC derivatives such as neural cells or glial cells after *in ovo* transplantation (Fig. 6C,D). On the other hand, recent direct conversion studies reported the incomplete silencing of reprogramming genes, indicating that the sustained expression of exogenous genes is necessary for the maintenance of the reprogrammed phenotype (Ieda et al., 2010; Sheng et al., 2012). It currently remains unknown whether the sustained expression of *Klf4* and/or c*-Myc* and/or *Sox10* is necessary to maintain converted SOX10+ NC cells in culture, and so strategies for conditionally induced expression of these three genes should be examined to clarify this point.

## MATERIALS AND METHODS

### Mice

*Sox10-IRES-Venus* mice (Motohashi et al., 2011) and *Sox1-Cre/+;Rosa26R-YFP/+* mice (Takashima et al., 2007) were maintained in our animal facility. *P0-promoter Cre/loxP-LacZ; Sox10-IRES-Venus* mice were generated by mating *Sox10-IRES-Venus* mice with *P0-promoter Cre/loxP-LacZ* ones (Yamazaki et al., 2005). The developmental stages of embryos were judged by their morphological appearance, as described in ‘The Mouse’ (Rugh, 1990). All animal experiments were performed in accordance with the Regulations of Animal Experiments in Gifu University.

### Flow cytometric analysis and cell sorting

E9.5 embryos of *Sox10-IRES-Venus* mice and *Sox1-Cre/+;Rosa26R-YFP/+* mice were incubated in 0.75 mg/ml collagenase (Wako) at room temperature for 20 min. After having been washed with PBS, each embryo was dissected in the region corresponding to the end of the branchial arches while being observed through a microscope (Carl Zeiss, DV4); and the cranial region was discarded. The dorsal region from somite containing neural tube, dorsal aorta, and notochord were extirpated from the trunk region of the embryos, incubated for 15 min at room temperature in Dispase II (Sanko-Jyunyaku), and then gently dissociated by passage through a 21-gauge needle. After washing with staining medium (SM: PBS containing 3% FSC), the cells were suspended in SM containing 3 µg/ml propidium iodide (PI: Calbiochem) to eliminate dead cells. For isolation of the converted SOX10+ cells, the infected fibroblasts were dissociated by incubating them for 6 min at 37°C in Dispase II (Sanko-Jyunyaku). After washing with SM, the cells were suspended in SM containing 3 µg/ml PI. All cell sorting and analyses were performed with a FACS Vantage dual-laser flow-cytometer (Becton-Dickinson). To analyze the expression of P75 with flow cytometry, cells were stained with anti-P75 (ab8875, Abcam) as the primary antibody and then with anti-rabbit IgG conjugated with DyLight 649 (Biolegend) as the secondary antibody.

### Microarray analysis and selection of 25 transcription factors

Total RNA was prepared as duplicate sample sets from isolated SOX10+ cells and SOX1+ cells by use of TRIZOL-LS Reagent (Invitrogen). SOX10+ cells and SOX1+ cells were sorted twice about 10,000 and 50,000 cells, respectively by FACS from E9.5 embryos. For the microarray experiments, total RNA prepared from SOX10+ cells were combined as one RNA sample due to duplicated labeled probes. The total RNA was labeled with Cy3-CTP and Cy5-CTP by using a Low RNA Input Fluorescent Linear Amplification Kit (Agilent), and hybridized with the NIA Mouse 44K Microarray v3.0 (Agilent, design NIA October 2008 best oligo). Each probe was normalized according to reference RNA signals (Carter et al., 2005). Microarray data were analyzed with NIA array analysis software ANOVA (NIH) and the Mouse Gene Index tool (Sharov et al., 2005a,b). Transcription factors were filtered by selecting genes with the ‘GO molecular function term’ ‘Transcription factor activity’ and ‘Transcription factor complex’. *In situ* expression patterns of the selected transcription factors were observed on the EMBRYS site (http://embrys.jp/embrys/html/MainMenu.html; (Yokoyama et al., 2009).

### Retroviral transfection and SOX10+ cell generation

Retrovirus vector pMXs-GW and pMYs-GW, in which the coding regions of 25 human transcription factors (Table 1) were cloned, was a gift from the human proteome expression resource (HuPEX) library (HuPEX clones in HGPD, http://www.HGPD.jp/, the National Institute of Advanced Industrial Science and Technology, Japan). Transfection of PLAT-E cells with the vectors was performed by using polyethyleneimine (Polysciences, Inc.). The PLAT-E cells were seeded at approximately 6.0-8.5×10^4^ cells/cm^2^ and transfected 20 h later. Individual supernatants containing the virus were harvested at 48 h post-transfection for infection. MEFs were seeded at 1.3×10^4^ cells/cm^2^, allowed to attach overnight, and were then infected with fresh retrovirus supplemented with polybrene (4 µg/ml, Nacalai Tesque) over 24 h. To achieve infection with 24 or three transcription factors, an equal volume of the retroviral supernatant was mixed before infection. The start of the virus infection period was termed ‘day 0’. After 24 h of infection, the medium was changed to the culture-supernatant of ST2 cell previously prepared. ST2 cells were maintained in α-MEM (Gibco) supplemented with 10% FCS, 10^−7^ M dexamethasone (Sigma), 20 pM bFGF (R&D Systems), 10 pM cholera toxin (Sigma), and 100 ng/ml human recombinant endothelin-3 (EDN3; Peptide Institute, Inc.) in 5% CO_2_ at 37°C. The culture-supernatant was collected after ST2 cells had been cultivated for two days in the above-mentioned medium.

### Maintenance and differentiation of SOX10+ cells

The converted SOX10+ cells were cultured under NC cell culture conditions based on those described by Morrison et al. (1999): a 5:3 mixture of DMEM-low:neurobasal medium (Gibco) supplemented with 15% Chick Embryo Extract (CEE, USBiological), 1% N2 (Gibco), 2% B27 (Gibco), 50 µM 2-mercaptoethanol (Sigma), 35 ng/ml all-trans retinoic acid (Sigma), 20 ng/ml IGF-1 (R&D systems), 100 ng/ml EDN3 (Peptide Institute. Inc.), and 20 ng/ml bFGF (R&D Systems). SOX10+ cells were incubated under hypoxic conditions with 5% O_2_ and 5% CO_2_ at 37°C (Hitachi, MCO-5M). After six days of cultivation under NC cell culture conditions, SOX10+ cells were differentiated into NC derivatives by using the following media: For differentiation into neural cells, glial cells or smooth muscle cells, SOX10+ cells were incubated in NC cell differentiation medium under hypoxic conditions at 37°C. The NC cell differentiation medium had the same components as the medium for NC cell culture conditions except that it contains 1% CEE, and 10 ng/ml bFGF (Morrison et al., 1999). For specific neural cell differentiation or glial cell differentiation, we incubated SOX10+ cells in NC cell differentiation medium supplemented with 50 ng/ml BMP-2 (R&D Systems), or in NC cell differentiation medium supplemented with 1 nM forskolin (Sigma) and 1 nM Nrg-1 (R&D Systems), respectively. For adipocyte differentiation, SOX10+ cells were incubated with adipogenic induction medium (LONZA). The medium was changed to adipogenic maintenance medium after three days and cells were then incubated for three more days. Four cycles of induction/maintenance were repeated. For osteocyte differentiation, we incubated SOX10+ cells in MSCGM medium (LONZA) supplemented with 0.1 µM Dex, 50 µg/ml ascorbate (Wako), and 0.1% β-glycerophosphate (Sigma). All differentiation cultures were performed under hypoxic conditions at 37°C.

### Immunohistochemical analysis

Colony fixation, permeabilization, and blocking were performed as described (Motohashi et al., 2014). Primary antibodies, diluted in 0.5% BSA PBS, were then added and allowed to react at room temperature. After having been washed with PBS, the cells were stained with the secondary antibodies in the same manner. Primary antibodies: anti-mouse neuronal class III β-tubulin (1:500; TuJ-1, Covance), anti-mouse glial fibrillary acidic protein (GFAP, 1:500; Z0334, DakoCytomation), anti-mouse α smooth muscle actin (1:500; 1A4, Sigma), anti-mouse peripherin (1:100; MAB1527, Chemicon), anti-nestin (1:500; Rat401, Chemicon), and anti-S100β (1:100, Sigma, SH-B1). Secondary antibodies: Texas Red-conjugated anti-mouse IgG (1:500; Molecular Probes) and Alexa Fluor 488-conjugated anti-rabbit IgG (1:500; Molecular Probes). Nuclei were stained with Hoechst 33258 (Sigma). Colonies were examined by using an Olympus IX-71 fluorescence microscope.

ALP staining was performed with an ALP staining kit (Muto Chemical Co.). Regarding Alizarin Red staining, cells were fixed in methanol at 4°C for 20 min, and Alizarin Red (Kanto Chemical) staining was then carried out for 5 min at room temperature. For Oil Red O staining, cells were fixed in 4% paraformaldehyde for 15 min and then stained with 60% Oil Red O solution (Wako) for 30 min at room temperature.

### Reverse transcription PCR and quantitative PCR analysis

Total RNA was purified by using Isogen (Nippon Gene), and first-strand cDNA synthesis was performed with Superscript III (Invitrogen). RT-PCR reactions were performed under the following conditions: 94°C, 2 min; 35-40 cycles of 94°C for 30 s, gene-specific annealing temperature for 30 s, and 72°C for 60 s. Quantitative PCR (qPCR) reactions were performed with Thermal Cycler Dice Real Time System (TAKARA) and SYBR premix Ex taq (TAKARA). The data were analyzed by the ΔΔCt method and plotted relative to β-actin expression. All primers used are described in Table S3.

### *In ovo* transplantation

The transplantation experiment was based on the procedures reported by Fernandes et al. (2004). Isolated SOX10+ cells were cultured under NC cell culture conditions, and were then collected six days later for transplantation. Cultured SOX10+ cells and MEFs were stained with the PKH26 Red Fluorescent Cell Linker (Sigma) following the manufacturer's instructions. The cells were injected by using a microinjector (IM-31, NARISHIGE) into the anterior, medial corner of 1 or 2 somites of each embryo, corresponding to the dorsal-most region of the neural-crest migratory pathway. The embryos were incubated for an additional three days to stage 29-30, fixed in 3.7% formaldehyde/PBS for 2 h, and subsequently immersed in 30% sucrose/PBS overnight at 4°C. The embryos were embedded in OCT (Sakura Finetechnical), sectioned at a 20-µm thickness, and placed on tissue-adhering slides. The slides were stained with TuJ-1 (1:50; BABCO) and anti- GFAP (1:50; Z0334, DakoCytomation), followed by Alexa Fluor 488-conjugated anti-mouse IgG (1:200; Molecular Probes) and Alexa Fluor 488-conjugated anti-rabbit IgG (1:200; Molecular Probes).

## References

[BIO015735C1] Amati B., Frank S. R., Donjerkovic D. and Taubert S. (2001). Function of the c-Myc oncoprotein in chromatin remodeling and transcription. *Biochim. Biophys. Acta* 1471, M135-M145. 10.1016/s0304-419x(01)00020-8 11250069

[BIO015735C2] Andrae J., Gallini R. and Betsholtz C. (2008). Role of platelet-derived growth factors in physiology and medicine. *Genes Dev.* 22, 1276-1312. 10.1101/gad.1653708 18483217 PMC2732412

[BIO015735C3] Baynash A. G., Hosoda K., Giaid A., Richardson J. A., Emoto N., Hammer R. E. and Yanagisawa M. (1994). Interaction of endothelin-3 with endothelin-B receptor is essential for development of epidermal melanocytes and enteric neurons. *Cell* 79, 1277-1285. 10.1016/0092-8674(94)90018-3 8001160

[BIO015735C4] Bellmeyer A., Krase J., Lindgren J. and LaBonne C. (2003). The protooncogene c-myc is an essential regulator of neural crest formation in xenopus. *Dev. Cell* 4, 827-839. 10.1016/S1534-5807(03)00160-6 12791268

[BIO015735C5] Belmadani A., Tran P. B., Ren D., Assimacopoulos S., Grove E. A. and Miller R. J. (2005). The chemokine stromal cell-derived factor-1 regulates the migration of sensory neuron progenitors. *J. Neurosci.* 25, 3995-4003. 10.1523/JNEUROSCI.4631-04.2005 15843601 PMC4461238

[BIO015735C6] Bitgood M. J. and McMahon A. P. (1995). Hedgehog and Bmp genes are coexpressed at many diverse sites of cell-cell interaction in the mouse embryo. *Dev. Biol.* 172, 126-138. 10.1006/dbio.1995.0010 7589793

[BIO015735C7] Black A. R., Black J. D. and Azizkhan-Clifford J. (2001). Sp1 and krüppel-like factor family of transcription factors in cell growth regulation and cancer. *J. Cell. Physiol.* 188, 143-160. 10.1002/jcp.1111 11424081

[BIO015735C8] Brambrink T., Foreman R., Welstead G. G., Lengner C. J., Wernig M., Suh H. and Jaenisch R. (2008). Sequential expression of pluripotency markers during direct reprogramming of mouse somatic cells. *Cell Stem Cell* 2, 151-159. 10.1016/j.stem.2008.01.004 18371436 PMC2276627

[BIO015735C9] Britsch S., Goerich D. E., Riethmacher D., Peirano R. I., Rossner M., Nave K.-A., Birchmeier C. and Wegner M. (2001). The transcription factor Sox10 is a key regulator of peripheral glial development. *Genes Dev.* 15, 66-78. 10.1101/gad.186601 11156606 PMC312607

[BIO015735C10] Cacalano G., Fariñas I., Wang L.-C., Hagler K., Forgie A., Moore M., Armanini M., Phillips H., Ryan A. M., Reichardt L. F. et al. (1998). GFRalpha1 is an essential receptor component for GDNF in the developing nervous system and kidney. *Neuron* 21, 53-62. 10.1016/S0896-6273(00)80514-0 9697851 PMC2710137

[BIO015735C11] Carreira S., Goodall J., Denat L., Rodriguez M., Nuciforo P., Hoek K. S., Testori A., Larue L. and Goding C. R. (2006). Mitf regulation of Dia1 controls melanoma proliferation and invasiveness. *Genes Dev.* 20, 3426-3439. 10.1101/gad.406406 17182868 PMC1698449

[BIO015735C12] Carter M. G., Sharov A. A., VanBuren V., Dudekula D. B., Carmack C. E., Nelson C. and Ko M. S. H. (2005). Transcript copy number estimation using a mouse whole-genome oligonucleotide microarray. *Genome Biol.* 6, R61 10.1186/gb-2005-6-7-r61 15998450 PMC1175992

[BIO015735C13] Cheung M. and Briscoe J. (2003). Neural crest development is regulated by the transcription factor Sox9. *Development* 130, 5681-5693. 10.1242/dev.00808 14522876

[BIO015735C14] Ding L., Saunders T. L., Enikolopov G. and Morrison S. J. (2012). Endothelial and perivascular cells maintain haematopoietic stem cells. *Nature* 481, 457-462. 10.1038/nature10783 22281595 PMC3270376

[BIO015735C15] Driskell R. R., Lichtenberger B. M., Hoste E., Kretzschmar K., Simons B. D., Charalambous M., Ferron S. R., Herault Y., Pavlovic G., Ferguson-Smith A. C. et al. (2013). Distinct fibroblast lineages determine dermal architecture in skin development and repair. *Nature* 504, 277-281. 10.1038/nature12783 24336287 PMC3868929

[BIO015735C16] Fan Y., Melhem M. F. and Chaillet J. R. (1999). Forced expression of the homeobox-containing gene Pem blocks differentiation of embryonic stem cells. *Dev. Biol.* 210, 481-496. 10.1006/dbio.1999.9279 10357905

[BIO015735C17] Fernandes K. J. L., McKenzie I. A., Mill P., Smith K. M., Akhavan M., Barnabé-Heider F., Biernaskie J., Junek A., Kobayashi N. R., Toma J. G. et al. (2004). A dermal niche for multipotent adult skin-derived precursor cells. *Nat. Cell Biol.* 6, 1082-1093. 10.1038/ncb1181 15517002

[BIO015735C18] Ghaleb A. M., Nandan M. O., Chanchevalap S., Dalton W. B., Hisamuddin I. M. and Yang V. W. (2005). Krüppel-like factors 4 and 5: the yin and yang regulators of cellular proliferation. *Cell Res.* 15, 92-96. 10.1038/sj.cr.7290271 15740636 PMC1317089

[BIO015735C19] Goseki-Sone M., Yamada A., Hamatani R., Mizoi L., Iimura T. and Ezawa I. (2002). Phosphate depletion enhances bone morphogenetic protein-4 gene expression in a cultured mouse marrow stromal cell line ST2. *Biochem. Biophys. Res. Commun.* 299, 395-399. 10.1016/S0006-291X(02)02646-3 12445813

[BIO015735C20] Graham A., Begbie J. and McGonnell I. (2004). Significance of the cranial neural crest. *Dev. Dyn.* 229, 5-13. 10.1002/dvdy.10442 14699573

[BIO015735C21] Griffiths I., Klugmann M., Anderson T., Thomson C., Vouyiouklis D. and Nave K. A. (1998). Current concepts of PLP and its role in the nervous system. *Microsc. Res. Tech.* 41, 344-358. 10.1002/(SICI)1097-0029(19980601)41:5<344::AID-JEMT2>3.0.CO;2-Q 9672418

[BIO015735C22] Hall B. K. (2009). *The Neural Crest and Neural Crest Cells in Vertebrate Development and Evolution*. New York: Springer.

[BIO015735C23] Han D. W., Tapia N., Hermann A., Hemmer K., Höing S., Araúzo-Bravo M. J., Zaehres H., Wu G., Frank S., Moritz S. et al. (2012). Direct reprogramming of fibroblasts into neural stem cells by defined factors. *Cell Stem Cell* 10, 465-472. 10.1016/j.stem.2012.02.021 22445517

[BIO015735C24] Hiramatsu K., Sasagawa S., Outani H., Nakagawa K., Yoshikawa H. and Tsumaki N. (2011). Generation of hyaline cartilaginous tissue from mouse adult dermal fibroblast culture by defined factors. *J. Clin. Invest.* 121, 640-657. 10.1172/JCI44605 21293062 PMC3026734

[BIO015735C25] Hong C.-S. and Saint-Jeannet J.-P. (2005). Sox proteins and neural crest development. *Semin. Cell Dev. Biol.* 16, 694-703. 10.1016/j.semcdb.2005.06.005 16039883

[BIO015735C26] Huang P., He Z., Ji S., Sun H., Xiang D., Liu C., Hu Y., Wang X. and Hui L. (2011). Induction of functional hepatocyte-like cells from mouse fibroblasts by defined factors. *Nature* 475, 386-389. 10.1038/nature10116 21562492

[BIO015735C27] Huleihel L., Ben-Yehudah A., Milosevic J., Yu G., Pandit K., Sakamoto K., Yousef H., LeJeune M., Coon T. A., Redinger C. J. et al. (2014). Let-7d microRNA affects mesenchymal phenotypic properties of lung fibroblasts. *Am. J. Physiol. Lung Cell. Mol. Physiol.* 306, L534-L542. 10.1152/ajplung.00149.2013 24441869 PMC3949080

[BIO015735C28] Ieda M., Fu J.-D., Delgado-Olguin P., Vedantham V., Hayashi Y., Bruneau B. G. and Srivastava D. (2010). Direct reprogramming of fibroblasts into functional cardiomyocytes by defined factors. *Cell* 142, 375-386. 10.1016/j.cell.2010.07.002 20691899 PMC2919844

[BIO015735C29] Khudyakov J. and Bronner-Fraser M. (2009). Comprehensive spatiotemporal analysis of early chick neural crest network genes. *Dev. Dyn.* 238, 716-723. 10.1002/dvdy.21881 19235729 PMC2650819

[BIO015735C30] Kim J., Lo L., Dormand E. and Anderson D. J. (2003). SOX10 maintains multipotency and inhibits neuronal differentiation of neural crest stem cells. *Neuron* 38, 17-31. 10.1016/S0896-6273(03)00163-6 12691661

[BIO015735C31] Kim Y. J., Lim H., Li Z., Oh Y., Kovlyagina I., Choi I. Y., Dong X. and Lee G. (2014). Generation of multipotent induced neural crest by direct reprogramming of human postnatal fibroblasts with a single transcription factor. *Cell Stem Cell* 15, 497-506. 10.1016/j.stem.2014.07.013 25158936

[BIO015735C32] Knecht A. K. and Bronner-Fraser M. (2002). Induction of the neural crest: a multigene process. *Nat. Rev. Genet.* 3, 453-461. 12042772 10.1038/nrg819

[BIO015735C33] Le Douarin N. M. and Kalcheim C. (1999). *The Neural Crest*. Cambridge: Cambridge University Press.

[BIO015735C34] Lemke G., Lamar E. and Patterson J. (1988). Isolation and analysis of the gene encoding peripheral myelin protein zero. *Neuron* 1, 73-83. 10.1016/0896-6273(88)90211-5 2483091

[BIO015735C35] Maclean J. A. II, Chen M. A., Wayne C. M., Bruce S. R., Rao M., Meistrich M. L., Macleod C. and Wilkinson M. F. (2005). Rhox: a new homeobox gene cluster. *Cell* 120, 369-382. 10.1016/j.cell.2004.12.022 15707895

[BIO015735C36] Manley N. R. and Capecchi M. R. (1995). The role of Hoxa-3 in mouse thymus and thyroid development. *Development* 121, 1989-2003. 7635047 10.1242/dev.121.7.1989

[BIO015735C37] Mansouri A., Pla P., Larue L. and Gruss P. (2001). Pax3 acts cell autonomously in the neural tube and somites by controlling cell surface properties. *Development* 128, 1995-2005. 11493522 10.1242/dev.128.11.1995

[BIO015735C38] McConnell B. B., Ghaleb A. M., Nandan M. O. and Yang V. W. (2007). The diverse functions of Krüppel-like factors 4 and 5 in epithelial biology and pathobiology. *Bioessays* 29, 549-557. 10.1002/bies.20581 17508399 PMC2211634

[BIO015735C39] Meulemans D. and Bronner-Fraser M. (2002). Amphioxus and lamprey AP-2 genes: implications for neural crest evolution and migration patterns. *Development* 129, 4953-4962. 12397104 10.1242/dev.129.21.4953

[BIO015735C40] Milet C., Maczkowiak F., Roche D. D. and Monsoro-Burq A. H. (2013). Pax3 and Zic1 drive induction and differentiation of multipotent, migratory, and functional neural crest in Xenopus embryos. *Proc. Natl. Acad. Sci. USA* 110, 5528-5533. 10.1073/pnas.1219124110 23509273 PMC3619367

[BIO015735C41] Mollaaghababa R. and Pavan W. J. (2003). The importance of having your SOX on: role of SOX10† in the development of neural crest-derived melanocytes and glia. *Oncogene* 22, 3024-3034. 10.1038/sj.onc.1206442 12789277

[BIO015735C42] Morrison S. J., White P. M., Zock C. and Anderson D. J. (1999). Prospective identification, isolation by flow cytometry, and in vivo self-renewal of multipotent mammalian neural crest stem cells. *Cell* 96, 737-749. 10.1016/S0092-8674(00)80583-8 10089888

[BIO015735C43] Motohashi T., Aoki H., Chiba K., Yoshimura N. and Kunisada T. (2007). Multipotent cell fate of neural crest-like cells derived from embryonic stem cells. *Stem Cells* 25, 402-410. 10.1634/stemcells.2006-0323 17038669

[BIO015735C44] Motohashi T., Yamanaka K., Chiba K., Miyajima K., Aoki H., Hirobe T. and Kunisada T. (2011). Neural crest cells retain their capability for multipotential differentiation even after lineage-restricted stages. *Dev. Dyn.* 240, 1681-1693. 10.1002/dvdy.22658 21594952

[BIO015735C45] Motohashi T., Kitagawa D., Watanabe N., Wakaoka T. and Kunisada T. (2014). Neural crest-derived cells sustain their multipotency even after entry into their target tissues. *Dev. Dyn.* 243, 368-380. 10.1002/dvdy.24072 24273191

[BIO015735C46] Nagai T., Aruga J., Takada S., Günther T., Spörle R., Schughart K. and Mikoshiba K. (1997). The expression of the mouse Zic1, Zic2, and Zic3 gene suggests an essential role for Zic genes in body pattern formation. *Dev. Biol.* 182, 299-313. 10.1006/dbio.1996.8449 9070329

[BIO015735C47] Ono N., Ono W., Mizoguchi T., Nagasawa T., Frenette P. S. and Kronenberg H. M. (2014). Vasculature-associated cells expressing nestin in developing bones encompass early cells in the osteoblast and endothelial lineage. *Dev. Cell* 29, 330-339. 10.1016/j.devcel.2014.03.014 24823376 PMC4083679

[BIO015735C48] Ouji Y., Ishizaka S., Nakamura-Uchiyama F. and Yoshikawa M. (2012). In vitro differentiation of mouse embryonic stem cells into inner ear hair cell-like cells using stromal cell conditioned medium. *Cell Death Dis.* 3, e314 10.1038/cddis.2012.56 22622133 PMC3366087

[BIO015735C49] Park K.-S. and Gumbiner B. M. (2010). Cadherin 6B induces BMP signaling and de-epithelialization during the epithelial mesenchymal transition of the neural crest. *Development* 137, 2691-2701. 10.1242/dev.050096 20610481 PMC2910385

[BIO015735C50] Rawadi G., Vayssière B., Dunn F., Baron R. and Roman-Roman S. (2003). BMP-2 controls alkaline phosphatase expression and osteoblast mineralization by a Wnt autocrine loop. *J. Bone Miner. Res.* 18, 1842-1853. 10.1359/jbmr.2003.18.10.1842 14584895

[BIO015735C51] Rugh R. (1990). *The Mouse*. New York: Oxford University Press.

[BIO015735C52] Sauka-Spengler T. and Bronner-Fraser M. (2008). A gene regulatory network orchestrates neural crest formation. *Nat. Rev. Mol. Cell Biol.* 9, 557-568. 10.1038/nrm2428 18523435

[BIO015735C53] Sekiya S. and Suzuki A. (2011). Direct conversion of mouse fibroblasts to hepatocyte-like cells by defined factors. *Nature* 475, 390-393. 10.1038/nature10263 21716291

[BIO015735C54] Sers C., Kirsch K., Rothbacher U., Riethmuller G. and Johnson J. P. (1993). Genomic organization of the melanoma-associated glycoprotein MUC18: implications for the evolution of the immunoglobulin domains. *Proc. Natl. Acad. Sci. USA* 90, 8514-8518. 10.1073/pnas.90.18.8514 8378324 PMC47387

[BIO015735C55] Sharov A. A., Dudekula D. B. and Ko M. S. H. (2005a). A web-based tool for principal component and significance analysis of microarray data. *Bioinformatics* 21, 2548-2549. 10.1093/bioinformatics/bti343 15734774

[BIO015735C56] Sharov A. A., Dudekula D. B. and Ko M. S. H. (2005b). Genome-wide assembly and analysis of alternative transcripts in mouse. *Genome Res.* 15, 748-754. 10.1101/gr.3269805 15867436 PMC1088304

[BIO015735C57] Sheng C., Zheng Q., Wu J., Xu Z., Wang L., Li W., Zhang H., Zhao X.-Y., Liu L., Wang Z. et al. (2012). Direct reprogramming of Sertoli cells into multipotent neural stem cells by defined factors. *Cell Res.* 22, 208-218. 10.1038/cr.2011.175 22064700 PMC3351918

[BIO015735C58] Shibata S., Yasuda A., Renault-Mihara F., Suyama S., Katoh H., Inoue T., Inoue Y. U., Nagoshi N., Sato M., Nakamura M. et al. (2010). Sox10-Venus mice: a new tool for real-time labeling of neural crest lineage cells and oligodendrocytes. *Mol. Brain* 3, 31 10.1186/1756-6606-3-31 21034515 PMC2989948

[BIO015735C59] Simoes-Costa M. and Bronner M. E. (2013). Insights into neural crest development and evolution from genomic analysis. *Genome Res.* 23, 1069-1080. 10.1101/gr.157586.113 23817048 PMC3698500

[BIO015735C60] Simoes-Costa M. and Bronner M. E. (2015). Establishing neural crest identity: a gene regulatory recipe. *Development* 142, 242-257. 10.1242/dev.105445 25564621 PMC4302844

[BIO015735C61] Simoes-Costa M., Tan-Cabugao J., Antoshechkin I., Sauka-Spengler T. and Bronner M. E. (2014). Transcriptome analysis reveals novel players in the cranial neural crest gene regulatory network. *Genome Res.* 24, 281-290. 10.1101/gr.161182.113 24389048 PMC3912418

[BIO015735C62] Simon C., Lickert H., Götz M. and Dimou L. (2012). Sox10-iCreERT2: a mouse line to inducibly trace the neural crest and oligodendrocyte lineage. *Genesis* 50, 506-515. 10.1002/dvg.22003 22173870

[BIO015735C63] Sridharan R., Tchieu J., Mason M. J., Yachechko R., Kuoy E., Horvath S., Zhou Q. and Plath K. (2009). Role of the murine reprogramming factors in the induction of pluripotency. *Cell* 136, 364-377. 10.1016/j.cell.2009.01.001 19167336 PMC3273494

[BIO015735C64] Srinivas S., Watanabe T., Lin C.-S., William C. M., Tanabe Y., Jessell T. M. and Costantini F. (2001). Cre reporter strains produced by targeted insertion of EYFP and ECFP into the ROSA26 locus. *BMC Dev. Biol.* 1, 4 10.1186/1471-213X-1-4 11299042 PMC31338

[BIO015735C65] Stemple D. L. and Anderson D. J. (1992). Isolation of a stem cell for neurons and glia from the mammalian neural crest. *Cell* 71, 973-985. 10.1016/0092-8674(92)90393-Q 1458542

[BIO015735C66] Stine Z. E., Huynh J. L., Loftus S. K., Gorkin D. U., Salmasi A. H., Novak T., Purves T., Miller R. A., Antonellis A., Gearhart J. P. et al. (2009). Oligodendroglial and pan-neural crest expression of Cre recombinase directed by Sox10 enhancer. *Genesis* 47, 765-770. 10.1002/dvg.20559 19830815 PMC2835405

[BIO015735C67] Takahashi M. and Osumi N. (2005). Identification of a novel type II classical cadherin: rat cadherin19 is expressed in the cranial ganglia and Schwann cell precursors during development. *Dev. Dyn.* 232, 200-208. 10.1002/dvdy.20209 15580626

[BIO015735C68] Takahashi K. and Yamanaka S. (2006). Induction of pluripotent stem cells from mouse embryonic and adult fibroblast cultures by defined factors. *Cell* 126, 663-676. 10.1016/j.cell.2006.07.024 16904174

[BIO015735C69] Takashima Y., Era T., Nakao K., Kondo S., Kasuga M., Smith A. G. and Nishikawa S.-I. (2007). Neuroepithelial cells supply an initial transient wave of MSC differentiation. *Cell* 129, 1377-1388. 10.1016/j.cell.2007.04.028 17604725

[BIO015735C70] Thier M., Wörsdörfer P., Lakes Y. B., Gorris R., Herms S., Opitz T., Seiferling D., Quandel T., Hoffmann P., Nöthen M. M. et al. (2012). Direct conversion of fibroblasts into stably expandable neural stem cells. *Cell Stem Cell* 10, 473-479. 10.1016/j.stem.2012.03.003 22445518

[BIO015735C71] Thompson C. B., Challoner P. B., Neiman P. E. and Groudine M. (1985). Levels of c-myc oncogene mRNA are invariant throughout the cell cycle. *Nature* 314, 363-366. 10.1038/314363a0 3982504

[BIO015735C72] Vallin J., Girault J.-M., Thiery J. P. and Broders F. (1998). Xenopus cadherin-11 is expressed in different populations of migrating neural crest cells. *Mech. Dev.* 75, 171-174. 10.1016/S0925-4773(98)00099-9 9739138

[BIO015735C73] Wahlbuhl M., Reiprich S., Vogl M. R., Bosl M. R. and Wegner M. (2012). Transcription factor Sox10 orchestrates activity of a neural crest-specific enhancer in the vicinity of its gene. *Nucleic Acids Res.* 40, 88-101. 10.1093/nar/gkr734 21908409 PMC3245941

[BIO015735C74] Wong C. E., Paratore C., Dours-Zimmermann M. T., Rochat A., Pietri T., Suter U., Zimmermann D. R., Dufour S., Thiery J. P., Meijer D. et al. (2006). Neural crest-derived cells with stem cell features can be traced back to multiple lineages in the adult skin. *J. Cell Biol.* 175, 1005-1015. 10.1083/jcb.200606062 17158956 PMC2064709

[BIO015735C75] Yamazaki H., Sakata E., Yamane T., Yanagisawa A., Abe K., Yamamura K.-I., Hayashi S.-I. and Kunisada T. (2005). Presence and distribution of neural crest-derived cells in the murine developing thymus and their potential for differentiation. *Int. Immunol.* 17, 549-558. 10.1093/intimm/dxh237 15837714

[BIO015735C76] Yokoyama S., Ito Y., Ueno-Kudoh H., Shimizu H., Uchibe K., Albini S., Mitsuoka K., Miyaki S., Kiso M., Nagai A. et al. (2009). A systems approach reveals that the myogenesis genome network is regulated by the transcriptional repressor RP58. *Dev. Cell* 17, 836-848. 10.1016/j.devcel.2009.10.011 20059953 PMC3110151

